# Cross-frequency coupling patterns during oddball processing in disorders of consciousness: a delta–gamma predominance

**DOI:** 10.3389/fpsyg.2025.1710742

**Published:** 2025-12-04

**Authors:** Binbin Huang, Qian Zhang, Hongwei Li, Qian Liu, Hongxing Cui, Deqiang Wang, Wei Li

**Affiliations:** 1 School of Special Education and Rehabilitation, Binzhou Medical University, Yantai, Shandong, China; 2 Department of Rehabilitation Medicine, Binzhou Medical University Hospital, Binzhou, Shandong, China

**Keywords:** disorders of consciousness, phase-amplitude coupling, oddball paradigm, delta oscillations, electroencephalography, task-specific oscillations

## Abstract

**Background:**

Phase–amplitude coupling (PAC) between low- and high-frequency oscillations provides a candidate mechanism for integrating information across cortical networks. While PAC alterations have been reported in disorders of consciousness (DOC), the frequency specificity and task dependence of these changes remain poorly characterized.

**Objective:**

To systematically examine cross-frequency coupling patterns during auditory oddball processing in DOC patients and explore whether specific frequency combinations or frequency shifts are associated with different levels of consciousness.

**Methods:**

Sixty-two participants [20 healthy controls (HC), 21 minimally conscious state (MCS), 21 unresponsive wakefulness syndrome (UWS)] underwent EEG recording during an auditory oddball paradigm. PAC was quantified using the Kullback–Leibler modulation index across comprehensive frequency ranges (phase: 1–31 Hz; amplitude: 8–77 Hz) without strong *a priori* band assumptions. Group comparisons relied on non-parametric permutation testing and bootstrap confidence intervals.

**Results:**

Delta–gamma coupling (1–4 Hz phase) dominated across all groups, with delta–gamma PAC consistently and significantly exceeding theta–gamma, alpha–gamma and beta–gamma coupling (all *p* < 0.001). A descriptive trend towards a downshift in the peak coupled amplitude frequency was observed along the consciousness continuum, with median values in the gamma range for HC (39.5 Hz) and MCS patients (35.0 Hz) and in the high-beta range for UWS patients (23.0 Hz), but this trend did not reach statistical significance (Kruskal–Wallis, *p* = 0.329). Conversely, between-group differences in delta–gamma coupling strength were not significant (*p* = 0.218), in the context of marked inter-individual heterogeneity (CV = 0.67 in HC, 0.73 in MCS) that was paradoxically lowest in the UWS group (CV = 0.50).

**Conclusion:**

Auditory oddball processing preferentially engages delta–gamma, rather than theta–gamma, coupling in both healthy individuals and DOC patients, supporting the view that PAC signatures are task- and paradigm-specific rather than universal markers of consciousness. Within this framework, a non-significant tendency towards frequency slowing and a reduction in PAC variability in UWS emerge as preliminary, hypothesis-generating features that warrant further investigation in larger, longitudinal and multi-paradigm cohorts.

## 1 Introduction

Disorders of consciousness (DOC) encompass a continuum of severe brain injuries in which awareness is partially or entirely lost, including unresponsive wakefulness syndrome (UWS) and the minimally conscious state (MCS) (Giacino et al., 2018). Accurate diagnosis within this spectrum remains a central challenge in clinical neuroscience (Gallucci et al., 2024). While the Coma Recovery Scale-Revised (CRS-R) remains the gold standard for behavioural assessment, its reliance on motor output introduces substantial diagnostic error (Bodien et al., 2023). Indeed, numerous studies have reported high misdiagnosis rates—30–40% of patients thought to be in UWS actually show signs of consciousness on specialized neuroimaging or electrophysiological tests (Li et al., 2025; Wang et al., 2020). This diagnostic uncertainty underscores the need for motor-independent neurophysiological markers that not only detect, but also better characterize residual neural processing (Diserens et al., 2023).

Phase-amplitude coupling (PAC) offers a promising neurophysiological approach for understanding consciousness-related neural dynamics (Borderie et al., 2024; Liang et al., 2021). This cross-frequency mechanism, whereby low-frequency oscillations modulate the amplitude of high-frequency activity, supports temporal coordination across distributed cortical networks (Hebscher et al., 2019; Roehri et al., 2022). While theta-gamma coupling has received considerable attention—with theta rhythms (4–7 Hz) orchestrating gamma bursts (30–100 Hz) during memory and sensory integration (Soltani Zangbar et al., 2020)—emerging evidence suggests that PAC patterns are highly task-dependent (Dong et al., 2022; Liu et al., 2022), For instance, Xiao et al. recently demonstrated enhanced theta-gamma coupling during music listening in MCS patients (Xiao et al., 2024). However, different cognitive paradigms may engage distinct oscillatory mechanisms, and the full spectrum of task-specific cross-frequency interactions in DOC populations remains systematically unexplored (King et al., 2013).

The auditory oddball paradigm provides a unique window into preserved attention and target detection mechanisms in DOC (Di et al., 2007; Bodien et al., 2024; Sharp et al., 2011). Given that delta oscillations coordinate large-scale cortical networks during attention-demanding tasks and that the P300 reflects delta phase resetting during target detection (Schnakers et al., 2008; Perrin et al., 2006; Laureys, 2005), oddball processing may engage different cross-frequency coupling patterns than those observed during continuous sensory stimulation (Laureys, 2005; Schiff, 2010; Bekinschtein et al., 2009). This raises the unexamined question of whether oddball detection preferentially engages delta-gamma rather than theta-gamma coupling (Demertzi et al., 2015).

Understanding task-specific oscillatory signatures has important implications for DOC assessment (Braiman et al., 2018). If different paradigms recruit distinct PAC patterns—theta-gamma for emotionally salient continuous stimuli versus other frequency combinations for discrete target detection—then comprehensive consciousness evaluation would require multiple complementary approaches rather than relying on a single biomarker (Perrin et al., 2015), Moreover, DOC populations exhibit considerable clinical and neurobiological heterogeneity that may manifest differently across various oscillatory mechanisms, potentially explaining inconsistent findings across studies (Thibaut et al., 2019).

To systematically investigate these questions, we characterized PAC patterns during auditory oddball processing in 62 individuals: 20 healthy controls (HC), 21 MCS patients, and 21 UWS patients. We employed a comprehensive, data-driven analytical approach, examining coupling across the full frequency spectrum (1–31 Hz for phase, 8–77 Hz for amplitude) without *a priori* constraints on specific frequency band combinations. This strategy enabled us to identify naturally occurring coupling patterns while accounting for the substantial inter-individual variability inherent to this clinical population. Given the cross-sectional design, we sought to characterize rather than establish causal relationships, recognizing that any observed differences may reflect consequences of impaired consciousness, compensatory mechanisms, or epiphenomena unrelated to awareness.

Our investigation addressed three key questions: First, which cross-frequency coupling patterns characterize oddball processing, and do these differ from previously reported patterns during other cognitive tasks? Second, what is the relationship between PAC patterns and consciousness levels? Specifically, do coupling strength, frequency characteristics, or spatial distribution vary systematically across diagnostic categories despite substantial biological heterogeneity? Third, are there frequency-specific alterations (such as oscillatory slowing or shifting) that might be associated with the severity of consciousness impairment? By addressing these questions without constraining our analysis to predetermined frequency bands, we aimed to provide an unbiased characterization of oddball-related oscillatory dynamics in disorders of consciousness, contributing to the development of task-specific neurophysiological assessment protocols.

Left panel: participant recruitment, eligibility screening, and group allocation (HC: healthy controls; MCS: minimally conscious state; UWS: unresponsive wakefulness syndrome). CRS-R assessments were conducted over 5 consecutive days with the highest score retained. Right panel: EEG signal processing pipeline including preprocessing, phase-amplitude coupling (PAC) analysis using Kullback–Leibler Modulation Index across 240 frequency pairs, band aggregation, and statistical testing. All groups were matched for age and sex (*p* > 0.05).

## 2 Methods

### 2.1 Participants and ethical considerations

This study was approved by the Ethics Committee of the Affiliated Hospital of Binzhou Medical University (Approval No. 2023-KT-110) and was conducted in strict accordance with the principles of the Declaration of Helsinki. Written informed consent was obtained from the legal guardians of all patients and directly from all HC prior to their inclusion in the study.

All participants were required to meet specific eligibility criteria. For patients, the inclusion criteria were: (a) age between 35 and 75 years; (b) a diagnosis of MCS or UWS; and (c) preserved auditory function, as verified by startle responses or review of medical records. The exclusion criteria were: (a) a history of epilepsy or other neurological conditions that could alter EEG patterns; (b) the presence of skull defects or major cranial wounds; and (c) severe agitation or involuntary movements likely to introduce significant artifacts into the recordings. While the protocol aimed to minimize pharmacological confounds, the ongoing use of psychoactive medications (e.g., anticonvulsants, benzodiazepines) deemed clinically necessary was not an absolute exclusion criterion. To address this potential confound, these medications were systematically documented (see Supplementary Table S1) and their influence was statistically evaluated in an exploratory analysis (see Results 3.5).

For HC, inclusion required an age between 35 and 75 years and no history of neurological or psychiatric disorders, while exclusion criteria included the use of any psychoactive medication.

Between November 2023 and January 2025, patients with a DOC who met these criteria were consecutively recruited from the Department of Rehabilitation Medicine. Age- and sex-matched HC were recruited from the local community. The diagnostic classification for each patient was rigorously confirmed using the CRS-R, in line with international standards. To ensure accuracy, two independent, certified examiners assessed each patient on five consecutive days; the highest score obtained during this period was used to establish the final diagnosis. Arousal level was assessed prior to EEG recording using the arousal subscale of the CRS-R to verify spontaneous or stimulus-induced eye opening. Only patients demonstrating sufficient arousal were included in the final analysis.

This recruitment and screening process yielded a final cohort of 62 participants: 21 patients with MCS, 21 with UWS, and 20 HC. The sample size for patient groups was determined based on previous EEG studies, which typically include 20–25 subjects per group to ensure adequate statistical power for group-level comparisons (Wang et al., 2022; Yu et al., 2022). The three groups were well-matched, with no significant differences in age (HC: 50.67 ± 15.77 years; MCS: 55.67 ± 16.67 years; UWS: 58.77 ± 17.60 years) or sex distribution (*p* > 0.05). Among the patient groups, there was no significant difference in mean disease duration (MCS: 8.82 ± 5.65 months; UWS: 7.43 ± 6.21 months; p > 0.05), and the distribution of etiologies (vascular, traumatic, anoxic) was comparable (p > 0.05). As expected, CRS-R scores were significantly higher in the MCS group (9.72 ± 3.21) than in the UWS group (4.65 ± 1.55). While etiology may influence neurophysiological features, the limited sample size precluded subgroup analyses. Detailed demographic and clinical characteristics are summarized in Table 1.

**Table 1:** Basic information of the study participants.

Group (N)	HC = 20	MCS = 21	UWS = 21	*P*
Sex (male/female)	10/10	13/8	12/9	>0.05
Age (years)	50.67 ± 15.77	55.67 ± 16.67	58.77 ± 17.60	>0.05
Vascular		12	13	
Traumatic		6	7	
Anoxic		3	1	
Disease duration, months		8.82 ± 5.65	7.43 ± 6.21	>0.05
CRS-R Score		9.72 ± 3.21	4.65 ± 1.55	–

CRS-R denotes the Coma Recovery Scale–Revised; MCS and UWS refer to the minimally conscious state and unresponsive wakefulness syndrome, respectively. No statistically significant differences were observed between the two groups (*p* > 0.05).

### 2.2 Experimental design and auditory stimulation

An auditory oddball paradigm was used to probe neural processing associated with conscious perception. Standard tones (1,000 Hz, 80%) and deviant tones (800 Hz, 20%) were presented in a pseudorandom order, with at least two standards preceding each deviant. A total of 1,000 stimuli were delivered, each lasting 200 ms (including 10 ms rise/fall times), and separated by interstimulus intervals of 400–600 ms. The overall session lasted about 11 min, with short breaks as needed.

Throughout data collection, vital signs (heart rate, blood pressure, respiration rate, and oxygen saturation) were monitored continuously. Recordings were paused if any value fell outside the predetermined safety range (heart rate < 50 or > 120 bpm; systolic BP < 90 or > 180 mmHg; respiration < 10 or > 25 per min; oxygen saturation < 92%). Stimulus generation and timing were controlled via MATLAB (R2022b) and PsychoToolbox v3.0.

### 2.3 EEG recording and preprocessing

EEG activity was collected using a 64-channel wireless recording system (NeuSen W, Neuracle, China) at a 1,000 Hz sampling rate. A subset of 22 electrodes (Fp1, Fp2, F7, F3, Fz, F4, F8, T7, C3, Cz, C4, T8, FC5, FC1, FCz, FC2, FC6, P7, P3, Pz, P4, P8) referenced to CPz was used for analysis. Electrode impedance was kept below 10 kΩ, and online filters included a 0.1–100 Hz band-pass and 50 Hz notch filter.

Preprocessing was performed using EEGLAB (Delorme and Makeig, 2004) (v2024.2) in MATLAB R2022b following standard procedures: (1) FIR band-pass filtering (1–100 Hz) and notch filtering (48–52 Hz); (2) downsampling to 500 Hz; (3) Interpolation of noisy or missing channels (average 2.3 ± 1.5 channels; no group difference, *p* = 0.65); (4) Epoch segmentation from −200 to 500 ms relative to stimulus onset; (5) Artifact removal using a ± 80 μV threshold and independent component analysis (ICA) with the ADJUST algorithm; (6) Average re-referencing; (7) Baseline correction within −200 to 0 ms.

### 2.4 PAC computation

Phase–amplitude coupling (PAC) was quantified using the Kullback–Leibler Modulation Index (KLMI) (Canolty and Knight, 2010; Tort et al., 2010), which measures deviations of amplitude distributions across phase bins from uniformity, providing a normalized and distribution-sensitive estimate of coupling strength that is less affected by amplitude variability than conventional indices (Tort et al., 2010).

To comprehensively map cross-frequency interactions, a band-to-band approach was employed (Onslow et al., 2011). Phase information was extracted from ten consecutive, non-overlapping 3 Hz-wide frequency bands spanning 1–4 Hz to 28–31 Hz, and amplitude information from twenty-four consecutive 3 Hz bands spanning 8–11 Hz to 74–77 Hz, yielding 240 frequency band-pair combinations per electrode.

For each band pair, signals were band-pass filtered using finite impulse response (FIR) filters defined by the corresponding frequency bounds. The Hilbert transform was applied to obtain instantaneous phase and amplitude envelopes. Phase values were binned into 18 equal intervals (−*π* to π), and the mean amplitude within each bin was computed to construct the empirical phase–amplitude distribution (*P*). The modulation index was calculated as

$$\mathrm{MI}=\frac{D_{\mathrm{KL}}(P,U)}{\log(N)},$$

Where $D_{\mathrm{KL}}\;\;$ represents the Kullback–Leibler divergence between the observed distribution (P) and a uniform distribution (U), and *N* = 18. This normalization constrains MI to the interval [0, 1], where 0 indicates no coupling and 1 indicates maximal coupling. Statistical significance was determined using surrogate analysis with 500 amplitude time-series block permutations; MI values exceeding the 95th percentile of the surrogate distribution were considered significant (*p* < 0.05).

To enable comparison with prior literature, we mapped frequencies to conventional bands: delta (1–4 Hz), theta (4–7 Hz), alpha (7–13 Hz), beta (13–30 Hz), and gamma (≥30 Hz). For statistical analyses, band-aggregated PAC metrics were obtained by averaging MI values across all constituent phase–amplitude combinations within each band pair (e.g., delta–gamma = phase 1–4 Hz × amplitudes ≥30 Hz). Where a 3-Hz amplitude bin straddled a boundary (e.g., 29–32 Hz), it was assigned to gamma to preserve the ≥30 Hz convention. Participant-level PAC was the median across 22 electrodes, which yielded robust estimates for group comparisons.

### 2.5 Statistical analysis

All statistical analyses were performed in Python 3.9 using the SciPy, NumPy, and pandas libraries (random seed = 2025 for reproducibility). As the data violated normality assumptions (Shapiro–Wilk test, *p* < 0.001), non-parametric methods were applied throughout. Within-group comparisons of PAC strength across frequency band pairs were conducted using Wilcoxon signed-rank tests, with rank-biserial correlation reported as the effect size. Between-group differences were assessed using permutation testing (10,000 iterations), while omnibus comparisons employed the Kruskal–Wallis H test. For post-hoc pairwise analyses, median differences were calculated and group labels were permuted 10,000 times to construct null distributions; *p*-values were estimated as (count + 1) / 10,001. Effect sizes were quantified using Cliff’s delta (*Δ*), interpreted according to standard thresholds (|Δ| < 0.147 negligible, 0.147–0.330 small, 0.330–0.474 medium, > 0.474 large). Multiple comparisons were controlled using the false discovery rate (FDR) procedure, and bootstrap 95% confidence intervals (5,000 iterations) were computed for group medians.

**Figure 1:**
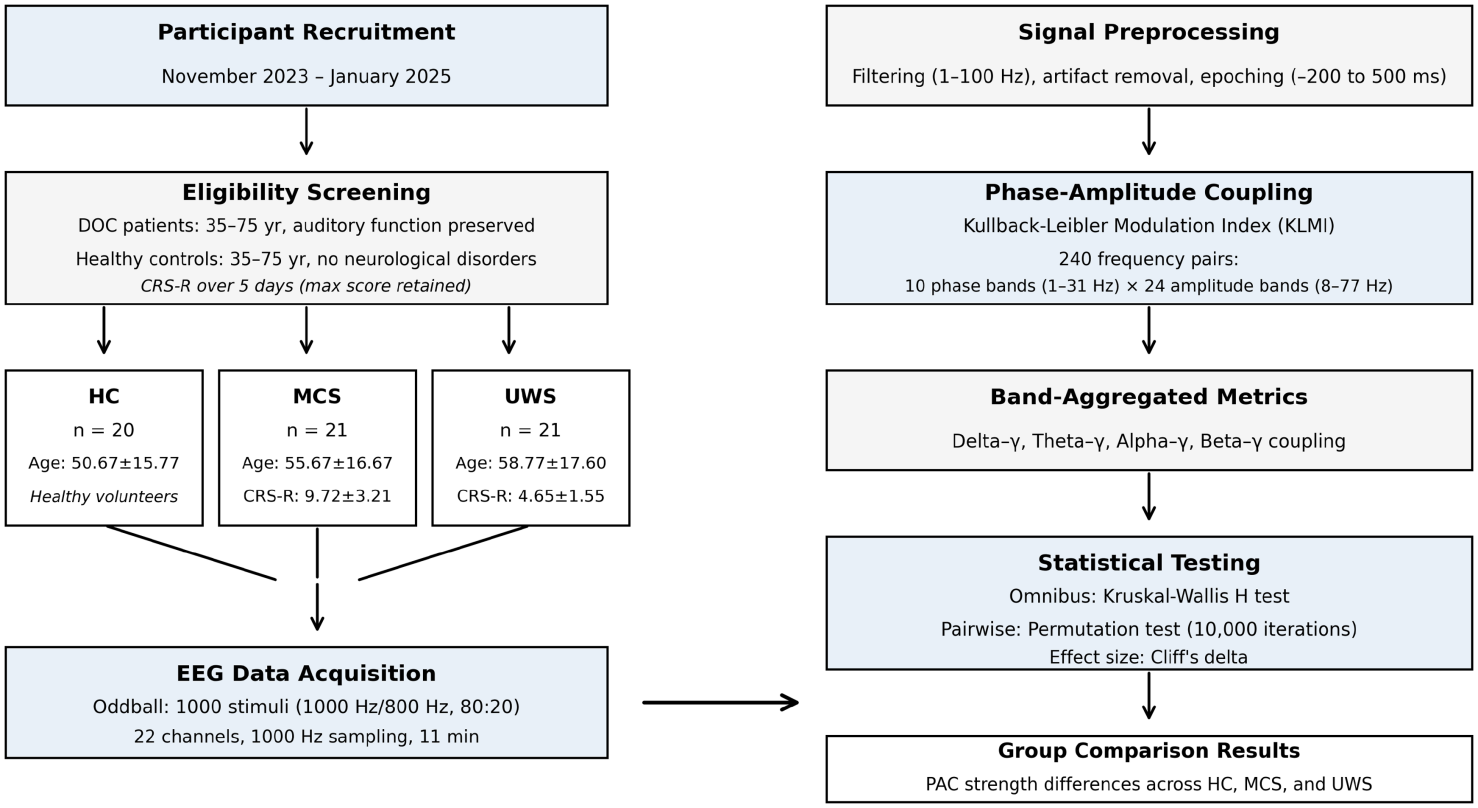
Study workflow and experimental design.

Within-group variability was expressed as the coefficient of variation (CV), and spatial differences in PAC strength were examined by comparing frontal and parietal electrode clusters (Wilcoxon signed-rank tests). Exploratory analyses evaluated associations between PAC metrics and clinical variables using Spearman’s rank correlation coefficient (*ρ*). Sedative medication load (0–4 scale) reflected the number of distinct CNS-depressant agents (benzodiazepines, GABA-B agonists, sedating anticonvulsants, or antipsychotics) administered within 24 h prior to EEG acquisition. Statistical significance was defined at *α* = 0.05, and all p-values and effect sizes are reported for transparency and reproducibility (see Figure 1).

## 3 Results

### 3.1 Delta–gamma coupling as the dominant PAC pattern across all groups

**Figure 2:**
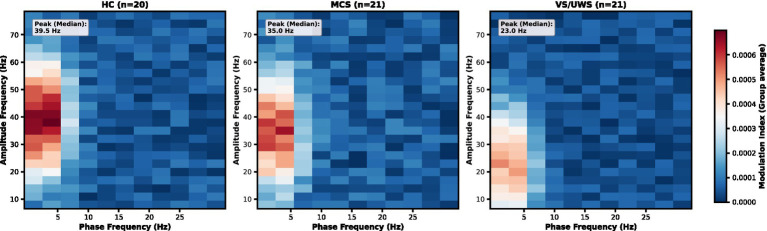
Frequency-specific phase-amplitude coupling patterns. Delta–gamma PAC strength across phase (1–31 Hz) and amplitude (8–77 Hz) frequencies for HC (*n* = 20), MCS (*n* = 21), and UWS (*n* = 21). The peak coupled amplitude frequency is labeled for each group (HC: 39.5 Hz, MCS: 35.0 Hz, UWS: 23.0 Hz). The heatmaps show group-averaged modulation index values across frequency pairs.

Systematic examination of all 240 frequency-pair combinations revealed that phase–amplitude coupling involving the delta frequency band (1–4 Hz phase) was the most prominent feature across all participant groups (Figure 2). To statistically confirm this observation, we compared the strength of delta–gamma PAC against other canonical frequency pairings (theta–gamma, alpha–gamma, and beta–gamma) within each group.

This analysis verified that delta–gamma coupling was significantly stronger than all other patterns. Specifically, Wilcoxon signed-rank tests confirmed that delta–gamma PAC strength was markedly higher than theta–gamma PAC in all groups: HC, Z = −3.78, *p* < 0.001, rank-biserial r = 0.82 (delta–gamma > theta–gamma); MCS, Z = −3.98, p < 0.001, r = 0.87; and UWS, Z = −3.92, p < 0.001, r = 0.85. Similar statistically significant dominance of delta–gamma coupling was observed when compared with alpha–gamma and beta–gamma coupling across all three groups (all *p* < 0.001).

**Figure 3:**
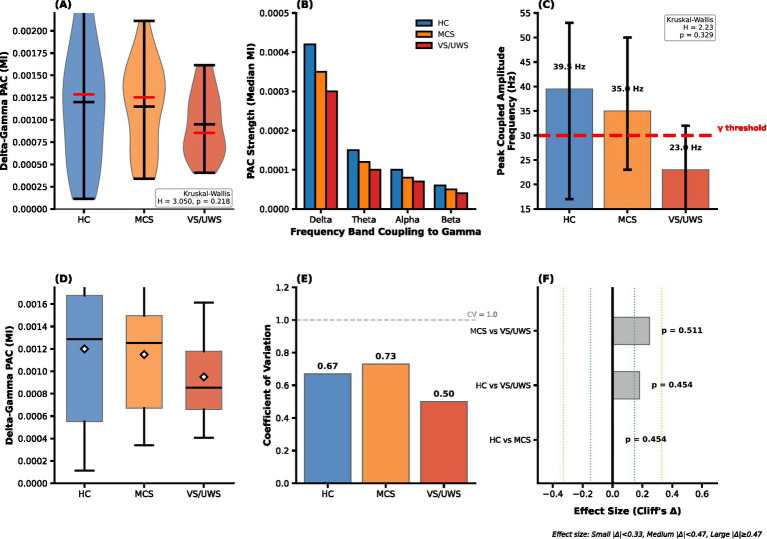
Phase-amplitude coupling during oddball processing in disorders of consciousness. **(A)** Primary Finding: Delta–gamma PAC strength across HC, MCS, and UWS groups. The Kruskal–Wallis test reveals no significant group differences (H = 3.050, *p* = 0.218). **(B)** Frequency Specificity: PAC strength is shown for delta, theta, alpha, beta, and gamma frequency bands.**(C)** Peak coupled amplitude frequency shows a descriptive downshift from low-gamma in HC (median 39.5 Hz) and MCS (35.0 Hz) to high-beta in UWS (23.0 Hz); Kruskal–Wallis: H = 2.23, *p* = 0.329 (n.s.). Red dashed line marks the gamma threshold (30 Hz; gamma defined as ≥30 Hz). **(D)** Individual Variability: Boxplots showing the variability of delta–gamma PAC strength across groups, with the outlier in HC highlighted in red. **(E)** Within-Group Heterogeneity: Coefficient of variation (CV) of delta–gamma PAC strength across groups. HC showed a CV of 0.67, MCS 0.73, and UWS 0.50. **(F)** Pairwise Comparisons: Effect sizes and *p*-values from permutation tests comparing delta–gamma PAC between groups, with no significant differences detected (all *p* > 0.4).

Visually, the magnitude of delta–gamma coupling was on average two- to threefold greater than that of other patterns (Figure 3B), underscoring its role as the primary cross-frequency interaction during the auditory oddball task.

### 3.2 Peak coupled amplitude frequency shifts from gamma to beta with impaired consciousness

Beyond overall coupling strength, we investigated whether the specific amplitude frequency that maximally coupled with the delta phase differed across groups. We observed a descriptive downshift of this peak coupled frequency along the continuum of consciousness (Figure 3C).

Specifically, the median peak coupled frequency was within the low-gamma band for HC (median = 39.5 Hz, IQR = 17.0–53.0 Hz) and MCS patients (median = 35.0 Hz, IQR = 23.0–50.0 Hz). In UWS, the peak shifted downward into the high-beta range (median = 23.0 Hz, IQR = 14.0–32.0 Hz), compared with low-gamma in HC and MCS.

However, this trend did not reach statistical significance (Kruskal–Wallis, *H*(2) = 2.23, *p* = 0.329; ε^2^ ≈ 0.018, small). This finding suggests a descriptive slowing of the coupled high-frequency oscillations with loss of consciousness. Here, “gamma” refers to amplitudes ≥30 Hz.

### 3.3 Spatial distribution of delta–gamma coupling is widespread

To examine the network-level organization of the dominant PAC pattern, we compared the strength of delta–gamma coupling between frontal (Fp1, Fp2, Fz, F3, F4, F7, F8) and parietal (Pz, P3, P4, P7, P8) electrode clusters.

**Figure 4:**
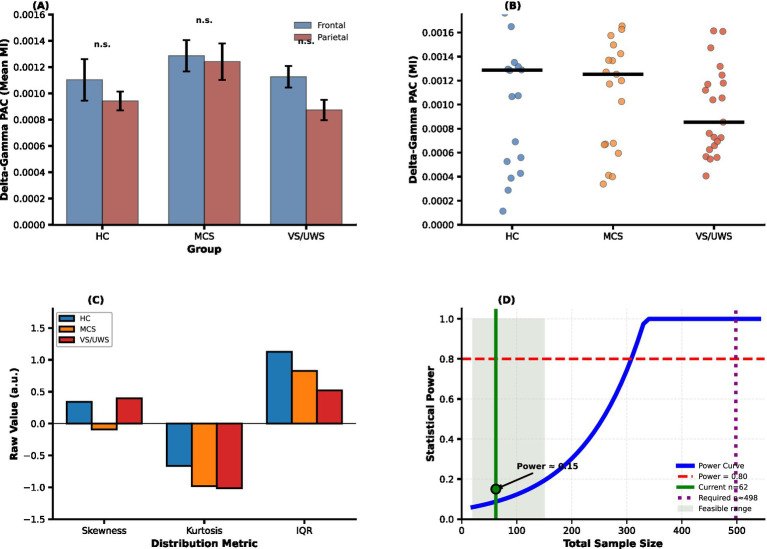
Analysis of spatial distribution, inter-individual variability, and statistical power. **(A)** Spatial Distribution Analysis (Within-Subject Paired Test): Delta–gamma PAC strength is compared between frontal and parietal electrode clusters for HC, MCS, and UWS groups. No significant differences were found across groups (n.s.). **(B)** Individual Subject Values (median): Median values for delta–gamma PAC strength across HC, MCS, and UWS, with individual subject data shown as circles and black horizontal lines indicating medians. **(C)** Distribution Parameters (Raw Values): Distribution metrics including skewness, kurtosis, and IQR for delta–gamma PAC strength in each group (HC, MCS, and UWS). **(D)** Post-hoc Power Analysis (ε^2^ = 0.018): The power curve demonstrates that the current sample size (*n* = 62) achieves approximately 15% power, with a required sample size of approximately 498 to achieve 80% power. The green shaded area shows the feasible range for recruitment.

No significant differences were detected between the two clusters in any group (HC: *p* = 0.956; MCS: *p* = 0.609; UWS: *p* = 0.838), indicating a widespread delta–gamma coupling distribution during the task (Figure 4A).

### 3.4 High inter-individual variability obscures between-group differences in PAC strength

#### 3.4.1 Quantification of within-group variability

Given the substantial inter-individual variability evident in the data distributions (Figure 3D), we formally quantified the variability of delta–gamma PAC within each group using the coefficient of variation (CV), defined as the ratio of the standard deviation to the mean. For completeness, per-subject median values and distribution-shape metrics are shown in Figures 4B,C, respectively.

The CV was high in both the HC (CV = 0.67) and MCS (CV = 0.73) groups, confirming pronounced heterogeneity in PAC strength across individuals. In contrast, variability was lowest in the UWS group (CV = 0.50) (Figures 3E, 4C).

#### 3.4.2 Between-group comparisons of PAC strength

Given the substantial intra-group variability, we next examined whether PAC strength could reliably distinguish between levels of consciousness using a robust permutation testing framework (10,000 permutations). For the primary outcome—delta–gamma coupling—we observed a descriptive trend toward decreasing median PAC strength from HC to UWS (Figure 3A). However, this trend did not reach statistical significance in the overall comparison (Kruskal–Wallis, *H*(2) = 3.050, *p* = 0.218). Pairwise permutation tests with FDR correction likewise revealed no significant group differences (HC vs. MCS: *p*₍FDR₎ = 0.454; HC vs. UWS: *p*₍FDR₎ = 0.454; MCS vs. UWS: *p*₍FDR₎ = 0.511). These pairwise comparisons are summarized in Figure 3F. The corresponding effect sizes were small (Cliff’s *Δ* for HC > UWS ≈ 0.27), suggesting that any true difference, if present, is modest and likely obscured by the considerable inter-individual variability (Figure 4D). To determine whether other coupling patterns might exhibit consciousness-related effects, we extended our analysis to theta–gamma coupling. Similar to the delta–gamma results, no significant differences were found among groups (permutation *p* = 0.344), and omnibus tests for theta–gamma, alpha–gamma, and beta–gamma PAC were likewise non-significant (all *p* > 0.30).

### 3.5 Exploratory analyses of confounding variables

To assess whether clinical factors influenced the observed PAC patterns, we conducted exploratory analyses within the patient cohort (n = 42) using the variables summarized in Supplementary Table S1 correlations between Sedative load (0–4) and either delta–gamma PAC or the peak coupled frequency were non-significant. The distribution of injury etiologies did not differ significantly between the MCS and UWS groups (Fisher’s exact test, *p* = 1.000), indicating comparable proportions of traumatic and non-traumatic causes. Across all patients, neither delta–gamma PAC strength nor the peak coupled frequency showed a significant correlation with time since injury (Spearman’s *ρ* = 0.009, *p* = 0.950; ρ = −0.061, *p* = 0.699, respectively). We also examined the potential impact of sedative medication load, calculated for each patient (see Methods), and again found no significant association with either delta–gamma PAC (ρ = −0.222, *p* = 0.155) or the peak coupled frequency (ρ = −0.012, *p* = 0.938). Although these analyses are exploratory and limited by statistical power, they provide no evidence that these clinical variables substantially contributed to—or confounded—the primary findings.

## 4 Discussion

This study systematically characterized PAC during an auditory oddball task in patients with DOC, revealing several key insights into their altered cortical oscillatory dynamics. Three main observations emerged: (1) delta–gamma coupling was the dominant cross-frequency interaction across all participant groups. (2) We observed a descriptive trend towards a downshift of the peak coupled amplitude frequency along the consciousness continuum, with median values shifting from the gamma range in HCs and MCS patients to the beta range in UWS patients, although this trend did not reach statistical significance (Kruskal–Wallis, *p* = 0.329). (3) Despite a descriptive trend of decreasing coupling strength, permutation tests did not reveal statistically significant group differences in delta–gamma PAC strength (*p* = 0.218). This null result occurred in the context of marked intra-group variability (CV = 0.67 in HC and 0.73 in MCS), which may have reduced power to detect modest between-group effects. Taken together, these findings suggest that PAC may not represent a universal signature of consciousness, but rather reflect task- and context-dependent oscillatory dynamics (Melloni et al., 2021).

The observed dominance of delta–gamma coupling—a finding that contrasts with many prior studies focused on theta–gamma (Tort et al., 2010)—warrants a detailed mechanistic consideration. This pattern is likely linked to the specific cognitive demands of the auditory oddball paradigm (Polich, 2007). The oddball task is the classic method used to elicit the P300 event-related potential (ERP), which is widely considered a marker of conscious target detection (Seth and Bayne, 2022). Crucially, the P300 itself is largely thought to be driven by, or reflected in, phase-resetting in the delta (1–4 Hz) band (Başar-Eroglu et al., 1992). Therefore, the delta–gamma coupling observed here may reflect the temporal coordination between this task-evoked slow delta potential (phase) and local gamma-band activity (amplitude) associated with cortical processing (Lakatos et al., 2005). This interpretation is also consistent with growing evidence that delta phase plays a key role in organizing the timing of sensory processing during attentional tasks (Schroeder and Lakatos, 2009).

Although the frequency downshift was not statistically significant (*p* = 0.329), the descriptive pattern of a lower peak coupled frequency in UWS compared to HC and MCS suggests a possible trend towards oscillatory slowing along the consciousness continuum. Given the exploratory nature of this effect and its limited statistical support, the following mechanistic considerations should be viewed as speculative, literature-based hypotheses rather than conclusions drawn directly from the present data. First, at the macro-circuit level, such slowing may reflect alterations in thalamocortical dynamics. According to mesocircuit models of consciousness (Schiff, 2010), the thalamus plays a central role in regulating cortical excitability and maintaining gamma oscillations. The apparent slowing in UWS could therefore be a downstream consequence of reduced thalamic drive, a hypothesis supported by neuropathological and imaging studies reporting preferential thalamic damage in DOC, particularly in UWS (Whyte et al., 2024). Second, at the micro-circuit level, a downshift in peak frequency may stem from disruption of local excitation–inhibition (E–I) balance, particularly involving fast-spiking PV + (parvalbumin-positive) interneurons. These high-energy-demand neurons are critical for pacing gamma rhythms; diffuse axonal injury or metabolic stress could selectively impair their function, thereby lengthening the oscillation cycle and lowering the peak frequency (Ruden et al., 2021). Finally, a shift towards lower frequencies might also represent an inefficient compensatory process (Guan et al., 2022). Slower oscillations are metabolically less costly, and a compromised brain network could be biased towards a more energy-efficient (but less functional) operational mode. Distinguishing whether such slowing primarily reflects irreversible damage, functional dysfunction, or adaptive compensation remains a key challenge for future research (Druga et al., 2023). In light of the non-significant group effect, our data should be regarded as providing preliminary, hypothesis-generating support for these mechanisms rather than definitive evidence.

Lesion maps (Supplementary Table S1) indicate that many patients had damage involving frontal and/or parietal association cortices and their connecting white matter, suggesting variable disruption of the frontoparietal network thought to support large-scale integration and cross-frequency coupling (Laureys et al., 2004). However, the marked heterogeneity and limited sample size precluded a robust subgroup analysis based on lesion topography, and we cannot draw firm conclusions about how specific lesion patterns modulate PAC in this cohort (Fernández-Espejo et al., 2011).

At first glance, the lack of significant group differences in PAC strength appears to diverge from influential studies that reported consciousness-related PAC alterations (Chennu et al., 2013). For example, prior work has demonstrated enhanced theta–gamma coupling in DOC patients during personalized music listening (Di Perri et al., 2016) or altered responses to a patient’s own name (Qin et al., 2010). Rather than representing a contradiction, this discrepancy highlights fundamental differences in task demands and stimulus salience (Naci et al., 2014). The theta–gamma alterations previously reported were driven by high-salience, emotionally and autobiographically meaningful stimuli (e.g., music, names) (Xiao et al., 2024). These complex stimuli recruit higher-order cognitive functions (e.g., memory, emotion) known to be impaired in UWS. In contrast, the present study used simple, non-personal, low-salience pure tones. Accordingly, our null result—the lack of group difference in delta–gamma strength—is compatible with the idea that more fundamental, automatic or pre-attentive sensory processing mechanisms may be partially preserved in UWS, even when higher-order processing indexed by theta–gamma coupling is compromised. Taken together, our findings and the existing literature support a key hypothesis: neural markers of consciousness are not “universal” but are highly dependent on the cognitive and affective system being engaged (Aru et al., 2019).

At the network level, our analysis of spatial distribution revealed a widespread and statistically equivalent (all *p* > 0.60) distribution of delta–gamma coupling across frontal and parietal clusters (Baars et al., 2013). This widespread, task-evoked pattern contrasts with the fronto-central predominance often seen in resting-state recordings (Nakatani et al., 2014), suggesting that oddball detection activates a distributed cortical network (Siebenhühner et al., 2020). However, this finding must be interpreted with caution. In our original manuscript, we speculated that this global distribution reflected the widespread cortical integration necessary for “conscious access” (Global Workspace Theory, GWT). This inference was flawed, as it failed to explain a key fact: this global distribution was preserved in UWS patients. If this activity truly represented the global broadcast of “conscious access,” we would expect it to collapse in the UWS group. A more cautious interpretation, therefore, is that this preserved global activation may reflect a more fundamental, automatic or pre-attentive stage of processing that can persist in UWS, rather than the later “conscious” broadcast described by GWT.

An important question is how our findings relate to theories proposing that consciousness relies not on specific frequencies, but on the brain’s capacity for flexible cross-frequency coordination (Hyafil et al., 2015). On this point, we must correct a key logical error from our original manuscript. We previously misinterpreted the *p* = 0.218 null result as “preservation” of coupling and claimed that this supported such theories. This was a logical contradiction; as the reviewers noted, if these theories are correct, loss of consciousness (UWS) should lead to a decrease in coordination, not preserved levels. A more rigorous and data-consistent interpretation is as follows: first, the p = 0.218 is a null result, plausibly influenced by the high variability (Hameroff, 2022) and limited statistical power we discussed (i.e., a plausible Type II error). Second, we did observe a non-significant descriptive trend of decreasing coupling strength (HC > MCS > UWS). This trend (rather than “preservation”), if substantiated by future, larger-scale studies, would provide stronger support for the hypothesis that impaired consciousness is linked to a reduced capacity for cross-frequency coordination (Nakatani et al., 2014).

Although our primary group comparison of PAC strength did not reach statistical significance, this study offers several important methodological contributions. First, the results demonstrate the critical need for data-driven (rather than solely hypothesis-driven) analytical strategies in clinical populations (King and Dehaene, 2014). Had we limited our analysis to the *a priori* theta–gamma band, the central finding of delta–gamma dominance would have been missed. Second, this study (in its revised form) adopted a more robust statistical framework. Facing non-normal data (Maris and Oostenveld, 2007) and pronounced variability (Goldfine et al., 2011), we used permutation testing and bootstrapping (Methods 2.5) to assess group differences, which is more robust than relying solely on traditional non-parametric tests (Sachs and Gabriel, 2016) and provides a more transparent characterization of the uncertainty around our null result. Finally, our detailed reporting of variability metrics (e.g., CV, IQR) (Rousselet et al., 2017) emphasizes the importance of reporting dispersion measures beyond group means, which is essential for future meta-analyses and for interpreting null effects. Although we did not implement a full Bayesian hierarchical framework, we explicitly highlight in the Limitations how future studies with larger samples could leverage Bayesian approaches to model uncertainty and heterogeneity more formally.

Several key limitations must be considered when interpreting these findings. First, the sample size (n ≈ 21 per group), while typical for DOC research (Lakens, 2013), provided adequate statistical power only for large effects. As discussed, the small-to-modest effect sizes observed mean that our null result for PAC strength may reflect limited power, a systemic challenge in the field (Wagenmakers et al., 2018). Second, the study’s cross-sectional design precludes causal inferences. We cannot determine whether the observed patterns—such as the descriptive trend of frequency downshift and the trend of decreasing strength (Andrade, 2025; Chennu et al., 2013)—represent a direct consequence of impaired consciousness, a compensatory mechanism, or an unrelated epiphenomenon (Aru et al., 2012). Finally, a major limitation is the uncontrollable clinical heterogeneity of the patient cohort. As detailed in Supplementary Table S1, patients show substantial diversity in structural lesion location (Fernández-Espejo et al., 2011). Furthermore, although our exploratory analysis (Results 3.5) found no significant effects, confounding variables such as sedative medication load and uncontrolled internal arousal fluctuations (Chennu et al., 2014) almost certainly contributed to the high intra-group variability observed.

## 5 Conclusion

In summary, this study provides a systematic characterization of phase–amplitude coupling during an auditory oddball task in disorders of consciousness. Across healthy controls and patients, delta–gamma coupling, rather than theta–gamma, emerged as the dominant cross-frequency interaction, underscoring that oscillatory signatures related to consciousness are strongly task-specific rather than universal. We observed a descriptive trend towards a downshift of the peak coupled frequency from the gamma to the beta range with decreasing consciousness level, but this effect did not reach statistical significance and should be considered preliminary. Group differences in coupling strength were likewise non-significant in the context of substantial inter-individual variability, which was paradoxically lowest in UWS. These findings highlight both the potential and the limitations of PAC-based markers in this population and point to the need for larger, multi-site and multi-paradigm studies, ideally with longitudinal follow-up, to clarify how task-dependent oscillatory dynamics relate to residual consciousness after severe brain injury.

## Data Availability

The raw data supporting the conclusions of this article will be made available by the authors, without undue reservation.
