# Supplementary material for: Cross-frequency coupling patterns during oddball processing in disorders of consciousness: a delta–gamma predominance
Source: Front Psychol. 2025 Dec 4;16:1710742. doi: 10.3389/fpsyg.2025.1710742 (PMC12711717; doi:10.3389/fpsyg.2025.1710742)
Supplement: Supplementary file 1 [file Table_1.DOCX]

**Supplementary Table S1. Individual Patient Demographics, Clinical Characteristics, and Medication Status**

| **Patient ID** | **Age** | **Sex** | **Etiology** | **Months** | **CRS-R total** | **Main Lesion Location** | **Lesion Category** | **Medications** | **Sedative load**  **(0–4)** | | **Classes counted** |
| --- | --- | --- | --- | --- | --- | --- | --- | --- | --- | --- | --- |
| MCS01 | 65 | M | Vascular | 12 | 12 | Left intracerebral hemorrhage | Cortical/Deep* | Amantadine | 0 |  | |
| MCS02 | 45 | F | Traumatic | 9 | 10 | Severe diffuse axonal injury | Diffuse | Levetiracetam | 0 |  | |
| MCS03 | 72 | M | Vascular | 7 | 9 | Brainstem hemorrhage, pontine | Brainstem | None | 0 |  | |
| MCS04 | 58 | M | Anoxic | 14 | 11 | Post-cardiac arrest anoxic encephalopathy | Diffuse | Amantadine, Baclofen | 1 | GABA-B | |
| MCS05 | 60 | F | Vascular | 10 | 13 | Right thalamic hemorrhage | Deep structures | None | 0 |  | |
| MCS06 | 40 | M | Traumatic | 15 | 8 | Multiple cerebral contusions | Cortical* | Topiramate | 1 | SAED | |
| MCS07 | 59 | F | Vascular | 4 | 10 | Multiple cerebral infarctions | Cortical* | Donepezil | 0 |  | |
| MCS08 | 50 | M | Traumatic | 6 | 11 | Frontal lobe hemorrhage | Cortical | None | 0 |  | |
| MCS09 | 68 | M | Vascular | 13 | 9 | Basal ganglia hemorrhage | Deep structures | Clonazepam | 1 | BZD | |
| MCS10 | 53 | F | Vascular | 16 | 14 | Intraventricular hemorrhage | Deep structures | Valproate | 1 | SAED | |
| MCS11 | 75 | M | Vascular | 3 | 8 | Cerebral hemorrhage | Cortical/Deep* | None | 0 |  | |
| MCS12 | 51 | F | Traumatic | 8 | 12 | Epidural hematoma, temporal | Cortical | Gabapentin | 1 | SAED | |
| MCS13 | 57 | M | Vascular | 5 | 10 | Multiple cerebral infarctions | Cortical* | Levetiracetam | 0 |  | |
| MCS14 | 64 | M | Vascular | 7 | 9 | Right parietal stroke | Cortical | Citalopram | 0 |  | |
| MCS15 | 42 | F | Traumatic | 11 | 11 | Severe DAI | Diffuse | Amantadine | 0 |  | |
| MCS16 | 56 | M | Vascular | 6 | 10 | Brainstem stroke | Brainstem | None | 0 |  | |
| MCS17 | 62 | F | Anoxic | 10 | 9 | Post-septic shock encephalopathy | Diffuse | Baclofen | 1 | GABA-B | |
| MCS18 | 48 | M | Vascular | 9 | 13 | Subdural hematoma | Cortical* | None | 0 |  | |
| MCS19 | 52 | F | Traumatic | 12 | 8 | Left MCA territory infarction | Cortical | Sertraline | 0 |  | |
| MCS20 | 66 | M | Vascular | 2 | 10 | Thalamic hemorrhage | Deep structures | Lamotrigine | 0 |  | |
| MCS21 | 54 | M | Anoxic | 8 | 11 | Cerebral hemorrhage | Cortical/Deep* | None | 0 |  | |
| UWS01 | 65 | F | Vascular | 15 | 6 | Post-cardiac arrest global anoxia | Diffuse | Baclofen | 1 | GABA-B | |
| UWS02 | 50 | M | Traumatic | 10 | 5 | Severe DAI, corpus callosum | Diffuse | None | 0 |  | |
| UWS03 | 72 | M | Vascular | 8 | 4 | Brainstem hemorrhage | Brainstem | Gabapentin | 1 | SAED | |
| UWS04 | 58 | F | Traumatic | 13 | 6 | Multiple cerebral contusions | Cortical* | Phenobarbital | 1 | SAED | |
| UWS05 | 75 | M | Vascular | 9 | 3 | Basal ganglia hemorrhage | Deep structures | None | 0 |  | |
| UWS06 | 60 | F | Vascular | 2 | 5 | Massive intracerebral hemorrhage | Cortical/Deep* | Tizanidine | 0 |  | |
| UWS07 | 45 | M | Vascular | 11 | 4 | Severe DAI | Diffuse | None | 0 |  | |
| UWS08 | 70 | F | Traumatic | 16 | 6 | Brainstem hemorrhage, bilateral | Brainstem | Baclofen | 1 | GABA-B | |
| UWS09 | 68 | M | Vascular | 3 | 3 | Multiple cerebral infarctions | Cortical* | Levetiracetam | 0 |  | |
| UWS10 | 80 | F | Vascular | 5 | 5 | Large MCA infarction | Cortical | None | 0 |  | |
| UWS11 | 55 | M | Traumatic | 8 | 4 | Brainstem hemorrhage | Brainstem | Clonazepam | 1 | BZD | |
| UWS12 | 63 | M | Vascular | 10 | 6 | Severe TBI, multiple contusions | Cortical* | None | 0 |  | |
| UWS13 | 49 | F | Traumatic | 4 | 3 | Traumatic intracranial hemorrhage | Cortical/Deep* | Valproate | 1 | SAED | |
| UWS14 | 73 | M | Traumatic | 17 | 5 | Brainstem hemorrhage | Brainstem | None | 0 |  | |
| UWS15 | 66 | F | Vascular | 7 | 4 | Thalamic hemorrhage with IVH | Deep structures | Gabapentin | 1 | SAED | |
| UWS16 | 52 | M | Vascular | 6 | 6 | Severe intracranial injury | Cortical* | None | 0 |  | |
| UWS17 | 77 | F | Vascular | 2 | 5 | Multiple infarctions, watershed | Cortical* | Baclofen | 1 | GABA-B | |
| UWS18 | 40 | M | Anoxic | 12 | 3 | Severe DAI, brainstem contusion | Diffuse | None | 0 |  | |
| UWS19 | 69 | M | Vascular | 1 | 4 | Large cerebral infarction | Cortical* | Levetiracetam | 0 |  | |
| UWS20 | 47 | F | Vascular | 9 | 6 | Post-septic encephalopathy | Diffuse | None | 0 |  | |
| UWS21 | 71 | M | Traumatic | 4 | 5 | Cerebral hemorrhage | Cortical/Deep* | Topiramate | 1 | SAED | |

**Abbreviations:** CRS-R, Coma Recovery Scale-Revised; DAI, Diffuse Axonal Injury; IVH, Intraventricular Hemorrhage; MCA, Middle Cerebral Artery; MCS, Minimally Conscious State; TBI, Traumatic Brain Injury; UWS, Unresponsive Wakefulness Syndrome.
**¹ Etiology:** Primary cause of brain injury, categorized as **Vascular** (ischemic or hemorrhagic stroke), **Traumatic** (traumatic brain injury), or **Anoxic** (anoxic-ischemic encephalopathy).
**² Months:** Time since injury (months).
**³ Main Lesion Location:** Lesion descriptions based on available clinical imaging reports (CT and/or MRI).
**⁴ Lesion Category:** Broad anatomical classification based on clinical descriptions. **Cortical**: lesions primarily affecting cortical regions; **Deep structures**: thalamus or basal ganglia; **Brainstem**: midbrain, pons, or medulla; **Diffuse**: diffuse axonal injury or global anoxic-ischemic injury. An asterisk () indicates uncertainty in precise anatomical localization (e.g., “Cortical/Deep”). For summary counts, ambiguous entries (e.g., “Cortical/Deep*”) were assigned to the category deemed more likely from the clinical report; sensitivity counts allocating them to the alternative category yielded the same qualitative conclusions.
**⁵ Medications:** Psychoactive medications administered within **24 h** of EEG recording. While the protocol aimed to minimize pharmacological confounds, ongoing use of clinically indicated agents (e.g., anticonvulsants, benzodiazepines) was not an absolute exclusion criterion; their potential influence was evaluated in an exploratory analysis (see Results 3.5).
**Sedative load (0–4):** Counts the number of distinct CNS-depressant classes administered within **24 h** (range 0–4): benzodiazepines, GABA-B agonists (e.g., baclofen), sedating antiepileptic drugs (e.g., valproate, phenobarbital, topiramate, gabapentin/pregabalin), and antipsychotics. Medications outside these classes (e.g., amantadine, donepezil, tizanidine, **selective serotonin reuptake inhibitors [SSRIs]**, levetiracetam, lamotrigine) are not counted.
**Lesion Distribution Summary:** Groups showed substantial within-group anatomical heterogeneity with broadly comparable distributions across categories. Such heterogeneity likely contributes to large within-group PAC variability (**HC≈0.67, MCS≈0.73, UWS≈0.50**), reducing power to detect between-group effects.
